# Challenges in Repurposing Drugs in COVID-19 Pandemic. Debating on Potential New Refinements

**DOI:** 10.3389/fphar.2020.559996

**Published:** 2020-10-28

**Authors:** Giorgio Frega, Andrea Palloni, Giuseppe Di Pasquale, Gioconda Saccoccio, Alessandro Rizzo, Elisabetta Poluzzi, Primiano Iannone, Giovanni Brandi

**Affiliations:** ^1^Department of Experimental, Diagnostic and Specialty Medicine, Sant'Orsola-Malpighi Hospital, University of Bologna, Bologna, Italy; ^2^Oncologia Medica, Azienda Ospedaliero-Universitaria di Bologna, Via Albertoni, Bologna, Italy; ^3^Division of Cardiology, Maggiore Hospital, Bologna, Italy; ^4^Department of Medicine, Azienda Unità Sanitaria Locale (AUSL) Bologna, Bologna, Italy; ^5^Pharmacology Unit, Department of Medical and Surgical Sciences, University of Bologna, Bologna, Italy; ^6^National Center for Clinical Excellence Healthcare Quality & Safety, Istituto Superiore di Sanità, Rome, Italy

**Keywords:** COVID-19, SARS-CoV-2, hydroxychloroquine, clinical trial, antivirals drugs

## Introduction

The recent worldwide spread of COVID-19 is the greatest pandemic in the last hundred years. At the end of August, approximately 24 million cases of the SARS-CoV-2 virus have been diagnosed worldwide, and over 800,000 people have died as a consequence (WHO COVID-19 Dashboard). A variety of drugs are currently under investigation that may prove potentially effective against the virus based on their pharmacodynamics (Sanders et al., 2020). Among them, some act by hampering the entrance of the virus in the host cells, others act with different intracellular antiviral mechanisms, eventually acting as immune-modulators or cytokine storm modulators. (Kupferschmidt and Cohen, 2020). This opinion pinpoints the potential risks deriving from repurposing some drugs, focusing on hydroxychloroquine, antivirals, and corticosteroids as prototypes, since their usage has become extremely widespread during the current pandemic breakthrough. Evidence of the clinical effectiveness of these drugs has been the subject of intense debate and adverse events have been reported in clinical controlled trials and clinical practice.

This study discusses these drugs as models and the potential risks deriving from the use of well-known compounds in the context of a different and unfamiliar disease. Likewise, we acknowledge that proving efficacy is harder than finding signals on plausible adverse effects. The pandemic scenario sadly obliged us to prescribe drugs relying on their putative efficacy without waiting for confirmative results, but the latter condition indicates that we should exercise caution and be alert to adverse events. In this setting, this opinion article reaffirms the need for properly drawn multi-centric clinical trials, stimulating debate on the potential new strategies that aim to generate high-quality evidence.

### Suggestions From the COVID-19 Pandemic

The quest for efficacious antiviral compounds has led to the investigation of many drugs that are already used in the treatment of different infectious diseases, such as the case of old antimalarials, various antivirals, and corticosteroids (Seeler et al., 1946; Marois et al., 2014; D’Alessandro et al., 2020).

Hydroxychloroquine (HCQ), as well as its precursor chloroquine, is an antimalarial drug also used for the treatment of autoimmune diseases such as systemic lupus erythematosus and rheumatoid arthritis (Rainsford et al., 2015). HCQ has been recently proposed as a promising agent for patients affected by COVID-19, in response to encouraging preclinical data (Liu et al., 2020) and the quite favorable toxicity profile of anti-malarial prophylactic (McChesney, 1983; Salako, 1984). Despite the lack of reliable data on the clinical efficacy, HCQ was widely used as an off-label treatment during the present pandemic outbreak and has been under evaluation in clinical trials, in either single-arm or controlled studies (Sanders et al., 2020).

Intriguingly, these antimalarial agents were historically proposed as antiarrhythmics, although they showed slight QT-prolongation, while arrhythmic deaths were not reported (Burn, 1950; Burrell and Martinez, 1958). However, their potential to induce cardiac conduction abnormalities, ranging from QT-prolongation to rare torsades de pointes or ventricular arrhythmias, has to be taken into account especially in patients with pre-existing or ongoing heart disease (Dixon et al., 2020).

Moreover, there are further concerns about drug interactions (Rajeshkumar et al., 2020). In this way, azithromycin, which was recently used in association with hydroxychloroquine during the COVID-19 pandemic, has been previously shown to slightly increase the risk of cardiovascular death (Ray et al., 2012) and ventricular arrhythmia (Trifirò et al., 2017). A preclinical investigation of this combo in Guinea Pigs seems to exclude the facilitation of electrical instability. (Dunne et al., 2007)

Changing the therapeutic indication of a drug could presumably modify the related safety profile. This hypothesis can gain a greater relevance dealing with the current viral infection in light of the non-negligible hearth tropism of SARS-CoV-2 (Huang et al., 2020; Inciardi et al., 2020).

Cardiac involvement occurs frequently during various viral diseases such as influenza A, including Spanish flu (Bratincsák et al., 2010). Furthermore, myocarditis was confirmed trough PCR detection in myocardial tissues of patients with viral infections (Engblom et al., 1983; Bowles et al., 2003).

Acute myocardial involvement, which is reflected by an elevation in cardiac biomarkers, is a common event among COVID-19 hospitalized patients, with a reported incidence of about 20% (Shi et al., 2020). However, the underlying pathological condition that triggers myocardial damage is not completely clear. In a 2009 study on a murine model, the authors showed that SARS-CoV-1 infection induces myocardial inflammation through the ACE2 system (Oudit et al., 2009). The same research group found viral SARS-CoV-1 RNA in 35% of human autopsied hearts (Oudit et al., 2009). In a small series of patients with COVID-19 and ST-segment elevation, a high prevalence of non-obstructive cardiac disease was found, suggesting the role of non-coronary related myocardial injury (Bangalore et al., 2020).

In this context, more accurate comprehension of the interaction between SARS-CoV-2 and the cardiovascular system could derive from pathological findings. An autoptic series on COVID-19 deceased patients seemed to confirm the tropism of SARS-CoV-2 for the myocardial tissue. The first case of biopsy-proven myocardial localization of SARS-CoV-2 viral particles in a patient presenting with cardiogenic shock was also recently published (Tavazzi et al., 2020).

Another report of four cases shows the presence of scattered individual cell myocyte necrosis in all the patients and macroscopic aspects of cardiomegaly with no sign of coronaropathy (Fox et al., 2020). The post-mortem analysis of two COVID-19 cases, performed by core biopsies, showed the presence of focal edema, interstitial fibrosis, and myocardial hypertrophy, however, the authors hypothesized that these findings as related to pre-existing conditions (Tian et al., 2020). The RT-PCR of one out of two cases was positive (Tian et al., 2020). Other authors reported the evidence of a few interstitial mononuclear inflammatory infiltrates in a case report (Xu et al., 2020). Lastly, a research team conducted a preclinical comparative study on macaques without detecting virus replication in the heart by RT-qPCR (Rockx et al., 2020), but further pathological confirmations are needed.

Eventually, all of these findings seem to suggest that COVID-19 patients could be more vulnerable to the proarrhythmic effect of QT prolongation (e.g. polymorphic ventricular tachycardia as well-known as torsades de pointes) (Doyen et al., 2020). In this respect, virus-induced myocardial structural damage (e.g. myocardial fibrosis) may facilitate ventricular arrhythmias through the well-known electrophysiological mechanism of re-entry electrical circuits.

A recent report showed an increase in sudden deaths in outpatients (Baldi et al., 2020). This scenario can be attributed to the clinical spectra of SARS-CoV-2 infection (Wadman et al., 2020), although the impact of other concomitant players, such as the decrease rate in hospitalization for non-COVID related symptoms, should not be excluded (De Filippo et al., 2020). Arrhythmias are one of the possible complications in patients hospitalized with SARS-CoV-2 related pneumonia (Wang et al., 2020), and probably induced by metabolic and physiologic sequelae such as hypoxia and electrolyte abnormalities characterizing the clinical course of COVID-19 infection. Furthermore, structural heart disease is present in a significant number of patients with COVID-19 infection. In a recent Wuhan series of 138 hospitalized patients, arrhythmias were reported as a common complication in over 15% and 40% of total and intensive care unit admitted population, respectively (Wang et al., 2020). The same authors reported the onset of acute cardiac injury in 7 % of patients (Wang et al., 2020). Several factors are known to contribute to an increased risk of torsades de pointes including female patients, structural heart disease, electrolyte disturbances, hepatic or renal failure, and concomitant QT-prolonging drugs. The hyperthermia also could be another contributor that turns down the antiarrhythmic threshold and can be associated with an increased arrhythmic risk (Pasquié et al., 2004). In this scenario, the interaction of either pre-existent or ad-hoc prescribed drugs with a putative impact on the virus-disrupted physiology should be investigated, taking also into account the molecular interfacing between virus and host cell proteins (Malle, 2020). Moreover, other critical factors often coexist in the elderly population such as polypharmacotherapy and comorbidities, which must be considered (Tisdale et al., 2014).

Preprint data about the safety profile of short course administration of hydroxychloroquine on COVID-19 patients seems to be reassuring, while a 30-day cardiovascular increased mortality rate has been reported for the combo hydroxychloroquine-azithromycin (Lane et al., 2020). Two other studies reported a higher risk of QTc prolongation for different dosages of chloroquine or hydroxychloroquine associated with azithromycin (Chorin et al., 2020; Borba et al., 2020). In the first article thereof, authors reported a severe increase in the qTC (>500 ms) in 11% of patients included in the cohort and the percentage rose to 18% of patients treated with high-dose hydroxychloroquine (Chorin et al., 2020). Two further studies reported similar pieces of evidence (Mercuro et al., 2020; Bessière et al., 2020). A recent French series on 11 patients reported one case of discontinuation due to QT-prolongation (Molina et al., 2020).

Furthermore, the first pieces of evidence on chloroquine or hydroxychloroquine efficacy have been non-conclusive and somewhat contradictory (Chen et al., 2020; Gautret et al., 2020; Magagnoli et al., 2020; Mahevas et al., 2020; Million et al., 2020; Molina et al., 2020; Yu et al., 2020). The results from larger trials were not yet published when these safety concerns emerged. While patients who are hospitalized or included in clinical trials could benefit from strict monitoring, concerns have emerged about the potential risks deriving from a non-monitored prescription of repurposed medications in the wider proportion of patients treated outside of a supervised setting. Even though the FDA gave an emergency authorization for the use of HCQ in COVID 19 patients, they later warned against its usage in such a context. The NIH and WHO halted clinical trials of hydroxychloroquine, given that there was a lack of clinical benefits in hospitalized patients (NIH halts clinical trial of hydroxychloroquine, 2020; Casey et al., 2020) (WHO discontinues hydroxychloroquine and lopinavir/ritonavir treatment arms for COVID-19). Additional controversies have emerged from focalized analyses of potential cardiac toxicities, and two papers have been retracted (Mehra et al., 2020).

Moving on from the cardiovascular system and deepening understanding of drug interactions, the relevance of hepatic cytochromes deserves special mention, in light of recent investigations on antiviral compounds such as the combination lopinavir/ritonavir. A randomized trial recently published on NEJM, along with other retrospective analysis, failed to show a benefit in COVID-19 hospitalized patients (Cao et al., 2020; Gao et al., 2020). Furthermore, the administration of lopinavir/ritonavir has been previously shown to interfere with the activity of liver cytochrome P450 enzymes (Yeh et al., 2006). In this context, the low propensity of SARS-CoV-2 to induce alteration of the hepatic function, at least in non-severe cases, may have had an impact on the absence of specific drug-related dysfunctions (Cao et al., 2020; Pawlotsky, 2020). In the meantime, Cao B. and coauthors have hinted at the relative safety of some cytochrome inducing or inhibiting compounds in COVID-19 patients (Cao et al., 2020). However, caution should be maintained in administrating these kinds of drugs, particularly when they are coupled with other liver-dependent metabolism drugs. In this regard, the previous information gathered in HIV patients, together with evidence of the propensity of SARS-CoV-2 for human systems and organs, could suggest comparable results. Similarly, previous studies on protease inhibitors, including an ACTT-1 trial of remdesevir, a nucleotide analog, showed good results in terms of safety (Beigel et al., 2020). The exclusion criteria of the study were substantial (e.g. AST or ALT 5 times the ULN and impaired renal function).

Finally, contrasting preliminary findings have also surfaced about the administration of corticosteroids. Initially, these drugs were not considered due to discouraging results and concerns about their immunosuppressive activities, but they have now been welcomed as potential allies against inflammatory storms, representing a breakthrough in research (Ledford, 2020; Li et al., 2020; Lu et al., 2020). This evidence and examples suggest that there is an overwhelming need for new solutions to minimize the controversies deriving from distinct case series.

## Discussion

The ongoing impact of this infectious outbreak of COVID-19, which does not yet have any proven effective therapies, means that there is an ongoing urgent search for effective therapies. Ethical standards of research cannot discount the caveat that patients need to be protected from the potential risk of new treatments, especially in pursuing an active vaccine. It is crucial to avoid loosening the ethical principles underlying biomedical research and the unsupervised adoption of treatments based on conflicting results or media pressure, even if in an emergency. In this regard, the research and studies discussed here present significant emergent issues, and important arguments that require scientific debate.

The clinical and preclinical data summarized above should encourage researchers and physicians to pay more attention to repurposing drugs either alone or in combination with other potentially interacting medications. This approach could guarantee better surveillance and management of the patients, limiting the incidence of rare but potentially lethal adverse events (e.g. by providing the carry out of serial EKG-tests and adjustment of electrolyte abnormalities). In line with this rationale, a significant contribution could derive from the drafting and application of shared guidelines (Giudicessi et al., 2020). Drawing inspiration from the Voluntary Harmonization Procedure (Krafft et al., 2012), we propose that a specific international entity should be established to coordinate the assessment and management of non-sponsored or urgently-needed investigation of approved compounds, examining potential remedies for orphan diseases. Bringing together proposals from different research teams via a central organization could facilitate a fast and rigorous evaluation of possible issues. Furthermore, by gathering patient data, simultaneously on a large scale, the central unit could solve problems in terms of time and scale. This may contribute to reaching new conditional approvals based on stronger data, and curtailing the occurrence of potential adverse events. This approach could be worthwhile not only in terms of the emergence of a pandemic but also in tackling the issues of repurposing drugs in other orphans/rare diseases. Another more practical approach could be to plan large-scale, pragmatic collaborative studies (Adaptive Trials) with institutional endorsements, such as by the NHS in the UK (which endorsed the RECOVERY trial - NCT04381936), WHO-endorsed SOLIDARITY (NCT04330690), the ACCORD platform also in the UK (Wilkinson et al., 2020), or ACTIV in the US.

The contingency to obtain quick results about the efficacy of a new or well-known compound in a completely unusual context should reinforce the need to create translational medicine research teams in which the expertise of various medical specialists can deeply intersect with that of preclinical researchers (e.g. pharmacologists, biologist, biotechnologist, biochemical and, physiologist).

Finally, our opinion is not aimed to restrict a prudent administration of available medicaments in the singular patients but to avoid the spreading of expectations without waiting for conclusive proof, even when working under time constraints. In the global networking era, early reports on the presumptive efficacy of drugs, medication, or other technologies can easily circulate and constitute an enormous advantage for the progress and the building of exciting “high-speed” research results. On the other hand, we cannot forget to thoroughly investigate by maintaining measures that guarantee the safety of drug prescriptions.

## Author Contributions

GB, GF, and AP conceived the article. GF, AP, GP, GS, AR, EP, PI, and GB wrote the first version of the manuscript and reviewed the final version. All authors contributed to the article and approved the submitted version.

## Conflict of Interest

The authors declare that the research was conducted in the absence of any commercial or financial relationships that could be construed as a potential conflict of interest.

## Abbreviations

COVID-19, COronaVIrus Disease; HCQ, Hydroxychloroquine; RCT, Randomized Clinical Trial; RT-qPCR, Real-Time – quantitative Polymerase Chain Reaction; SARS-CoV-1, Severe Acute Respiratory Syndrome - Coronavirus – 1; SARS-CoV-2, Severe Acute Respiratory Syndrome - Coronavirus - 2.
